# Machine learning reduced workload for the Cochrane COVID-19 Study Register: development and evaluation of the Cochrane COVID-19 Study Classifier

**DOI:** 10.1186/s13643-021-01880-6

**Published:** 2022-01-22

**Authors:** Ian Shemilt, Anna Noel-Storr, James Thomas, Robin Featherstone, Chris Mavergames

**Affiliations:** 1 EPPI Centre, UCL Social Research Institute, University College London, 18 Woburn Square, London, WC1H 0NR UK; 2 Radcliffe Department of Medicine, University of Oxford, Level 4, John Radcliffe Hospital, Headington, Oxford, OX3 9DU UK; 3 Editorial & Methods Department, Cochrane, St Albans House, 57-59 Haymarket, London, SW1Y 4QX UK; 4 Informatics & Technology Services, Cochrane, St Albans House, 57-59 Haymarket, London, SW1Y 4QX UK

**Keywords:** Machine learning, Study classifiers, Searching, Information retrieval, Methods/methodology, Systematic reviews, Automation, Crowdsourcing, Cochrane Library, COVID-19

## Abstract

**Background:**

This study developed, calibrated and evaluated a machine learning (ML) classifier designed to reduce study identification workload in maintaining the Cochrane COVID-19 Study Register (CCSR), a continuously updated register of COVID-19 research studies.

**Methods:**

A ML classifier for retrieving COVID-19 research studies (the ‘Cochrane COVID-19 Study Classifier’) was developed using a data set of title-abstract records ‘included’ in, or ‘excluded’ from, the CCSR up to 18th October 2020, manually labelled by information and data curation specialists or the Cochrane Crowd. The classifier was then calibrated using a second data set of similar records ‘included’ in, or ‘excluded’ from, the CCSR between October 19 and December 2, 2020, aiming for 99% recall. Finally, the calibrated classifier was evaluated using a third data set of similar records ‘included’ in, or ‘excluded’ from, the CCSR between the 4th and 19th of January 2021.

**Results:**

The Cochrane COVID-19 Study Classifier was trained using 59,513 records (20,878 of which were ‘included’ in the CCSR). A classification threshold was set using 16,123 calibration records (6005 of which were ‘included’ in the CCSR) and the classifier had a precision of 0.52 in this data set at the target threshold recall >0.99. The final, calibrated COVID-19 classifier correctly retrieved 2285 (98.9%) of 2310 eligible records but missed 25 (1%), with a precision of 0.638 and a net screening workload reduction of 24.1% (1113 records correctly excluded).

**Conclusions:**

The Cochrane COVID-19 Study Classifier reduces manual screening workload for identifying COVID-19 research studies, with a very low and acceptable risk of missing eligible studies. It is now deployed in the live study identification workflow for the Cochrane COVID-19 Study Register.

## Background

The COVID-19 pandemic has resulted in an unprecedented level of article publications [1, 2] of which only a small percentage report study data or analytics [3]. This presented the systematic review community with significant challenges to identify and classify relevant study evidence reliably, accurately, and efficiently, to enable the rapid synthesis and use of cumulative bodies of evidence to inform international, national and local responses to the evolving global health crisis.

As the pandemic took hold, a number of initiatives were started with the aim of identifying and classifying COVID-19 research. Two such initiatives are the COVID-19 Open Research Dataset (CORD-19) developed by the Semantic Scholar Team at the Allen Institute [4] and COVID-19 L·OVE by Epistemonikos [5]. Each initiative had variable aims and different approaches to collating the required information; but, to our knowledge, the Cochrane COVID-19 Study Register (CCSR) was the only product designed to support rapid evidence synthesis through the identification and classification of ongoing and completed primary studies. Cochrane was able to utilise existing technical infrastructure, processes and human resource to create an open access register of COVID-19 studies. The Cochrane COVID-19 Study Register (CCSR) [6] includes primary, human studies across a broad range of areas relevant to COVID-19, including the treatment and management of the virus, diagnosis, prognosis, transmission and prevention, mechanism, epidemiology and the wider impact of the pandemic on populations and health services. The CCSR study records are validated and maintained by a team of Cochrane information and data curation specialists. Automated searches retrieve results via daily or weekly API calls across a range of sources. The results are then de-duplicated and screened. A sub-set of results (those retrieved from Embase) are sent to Cochrane Crowd, Cochrane’s citizen science platform [7]; the rest are screened by the core register team [8, 9]. The screening process involves an assessment of record eligibility based on titles and abstracts. For records without abstracts, more information is sought before a judgement is made. Eligible studies are then tagged by the team or by the Crowd according to study type, study design and study aims. Intervention studies are also annotated according to their PICO (population, intervention, comparator and outcome) components. These tagging and annotation activities, together with the largely manual process of linking related reports together, are resource intensive.

In July 2020, we convened a series of meetings between the CCSR team and the team from the EPPI Centre (UCL) and Centre for Reviews and Dissemination (University of York), which has been maintaining a living map of COVID-19 research evidence (the ‘C-19 living map’) commissioned by the UK Department of Health and Social Care. The purpose of these meetings was to share best practice and reduce duplication of effort between the respective workflows being used to keep these two overlapping resources up to date; and we have initially focused on strategies to reduce manual screening burden in the selection of eligible articles.

As the rate of COVID-19 publishing shows little sign of slowing, introducing machine learning (ML) into COVID-19 study identification workflows could offer important gains in terms of workload reduction [10] so long as the corollary risk of ‘missing’ (or ‘losing’) relevant research studies is acceptably low. The C-19 living map team had recently developed and deployed a ML classifier for this purpose, and similar classifiers have previously been deployed in Cochrane’s Centralised Search Service and Screen4Me workflows, to support efficient identification of randomised controlled trials (RCTs) [11].

For both the CCSR and the C-19 living map, we decided to deploy a ML classifier to discard records scoring below an identified threshold score, calibrated to minimise the risk of ‘missing’ eligible articles. However, given differences between the respective scopes and eligibility criteria of these two resources, we decided that a new binary ML classifier should be specifically developed for the CCSR workflow.

## Methods

In this study, we aimed to train (Stage 1), calibrate (Stage 2) and evaluate (Stage 3) a binary ML classifier (‘the classifier’) designed to reduce study identification workload in maintaining the CCSR, with an acceptably low corollary risk of ‘missing’ records of ‘included’ (eligible) studies. We therefore needed to assemble three separate data sets from the CCSR screening workflows (see below and ‘Availability of data and materials’).

### Training (Stage 1)

In Stage 1, we assembled a training data set containing bibliographic title-abstract records of all articles manually screened for eligibility for the CCSR from its first search date (March 20, 2020) up until October 18, 2020. Embase.com records had only been recently added to the CCSR’s sources by mid-October, and a backlog of medRxiv preprints was still being processed. As the CCSR’s other sources were trial registers (not bibliographic title-abstract records), most of the training set records were from PubMed. These records had originally been identified using conventional Boolean searches of selected electronic bibliographic databases and trials registries, before being manually screened and labelled as either ‘included’ (eligible for the CCSR) or ‘excluded’ (ineligible) by Cochrane information specialists or the Cochrane Crowd [7]. The search strategies used can be seen on the *About* page of the CCSR [6]. After removing trials registry records, we were left 59,513 records, of which 20,878 were labelled as ‘included’ in the CCSR, and 38,635 were ‘excluded’. These records were imported into *EPPI-Reviewer* [12], assigned to code sets and used to train a logistic regression classifier using tri-gram ‘bag of words’ features, implemented in the SciKit-Learn python library, with ‘included’ records designated as the positive class (class of interest) and ‘excluded’ records as the negative class.

### Calibration (Stage 2)

In Stage 2, we assembled a calibration data set containing 16,123 similar records manually screened for eligibility for the CCSR between the 19th October and 2nd of December 2020, again labelled as ‘included’ (6005 eligible records) or ‘excluded’ (10,118 ineligible records) by the same people and process, and with trials registry records having again been removed. The records were imported into *EPPI-Reviewer*, assigned to code sets and used to calibrate the classifier developed in Stage 1. Specifically, we applied the classifier to 16,123 calibration records, which automatically assigned a score (0–100) to each record. We then computed the threshold score that captured >99% of the ‘included’ records in this data set (i.e. recall >0.99). 0.99 is the threshold level of recall that is currently required for ML classifiers to be deployed in Cochrane systems and workflows [13]. We also computed standard performance metrics, namely: (cumulative) recall, (cumulative) precision and net workload reduction.

### Evaluation (Stage 3)

In Stage 3, we assembled an evaluation data set of similar records containing 4722 records manually screened for eligibility for the CCSR between the 4th and 19th of January 2021, once again labelled as ‘included’ (2310 eligible records) or ‘excluded’ (2412 ineligible records), with trials registry records removed. The records were imported into *EPPI-Reviewer*, assigned to code sets, and used to evaluate the classifier developed in Stage 1. Specifically, we applied the classifier to 4722 evaluation records, identified ‘included’ and ‘excluded’ records scoring above and below the threshold score we had computed in Stage 2, and then, we computed (cumulative) recall, (cumulative) precision and net workload reduction. We also analysed characteristics of ‘included’ articles that would have been ‘missed’ by the workflow if the classifier had been implemented.

Finally, we compared key characteristics of articles between the three study data sets described above in this section (training, calibration, evaluation), to check post hoc that they comprised similar enough sets of records to validate our results from calibrating and evaluating the classifier.

## Results

### Calibration

**Fig. 1:**
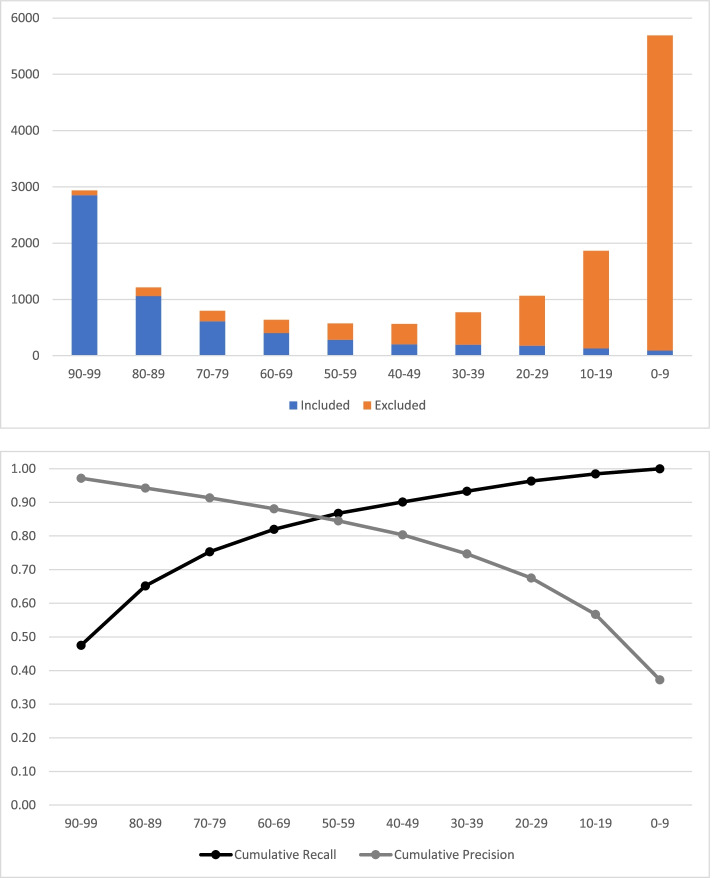
Distribution of classifier scores among ‘included’ and ‘excluded’ calibration records (*N*=16,123) and related performance metrics

Results from calibrating the Cochrane COVID-19 Study Classifier (Stage 2) are shown in Fig. 1 and Table 1. The threshold classifier score at target recall >0.99 was identified as 7 (Table 1), which means that >99% of ‘included’ records in the calibration set scored 7 or above. In this data set, retaining records scoring 7 or above, to achieve target recall >0.99 among ‘included’ records, would have resulted in an overall workflow precision of 0.52, with a corollary 29.1% reduction in manual screening workload.

**Table 1:** Distribution of classifier scores among ‘included’ and ‘excluded’ calibration records and related performance metrics

Classifier score	90–99	80–89	70–79	60–69	50–59	40–49	30–39	20–29	10–19	0–9	Totals
**Included** ***N***	2853	1059	610	402	284	202	195	180	129	91	6005
**Excluded** ***N***	83	156	190	237	290	364	578	885	1736	5599	10,118
**Totals**	2936	1215	800	639	574	566	773	1065	1865	5690	16,123
**Precision**	0.97	0.87	0.76	0.63	0.49	0.36	0.25	0.17	0.07	0.02	
**Cumulative recall**	0.48	0.65	0.75	0.82	0.87	0.90	0.93	0.96	0.98	1.00	
**Cumulative precision**	0.97	0.94	0.91	0.88	0.84	0.80	0.75	0.68	0.57	0.37	
**Threshold classifier score (recall >0.99)**	7										
**Screened included** ***N***^a^	5950										
**Screened excluded** ***N***^a^	5487										
**Precision**^a^	0.52										
**Discarded (‘Lost’) included** ***N***^a^	55										
**Discarded excluded** ***N***^a^	4631										
**Net workload reduction** ***N***^a^	4686										
**Net workload reduction %**^a^	29.1%										

^a^At threshold score = 7 (recall >0.99)

### Evaluation

**Fig. 2:**
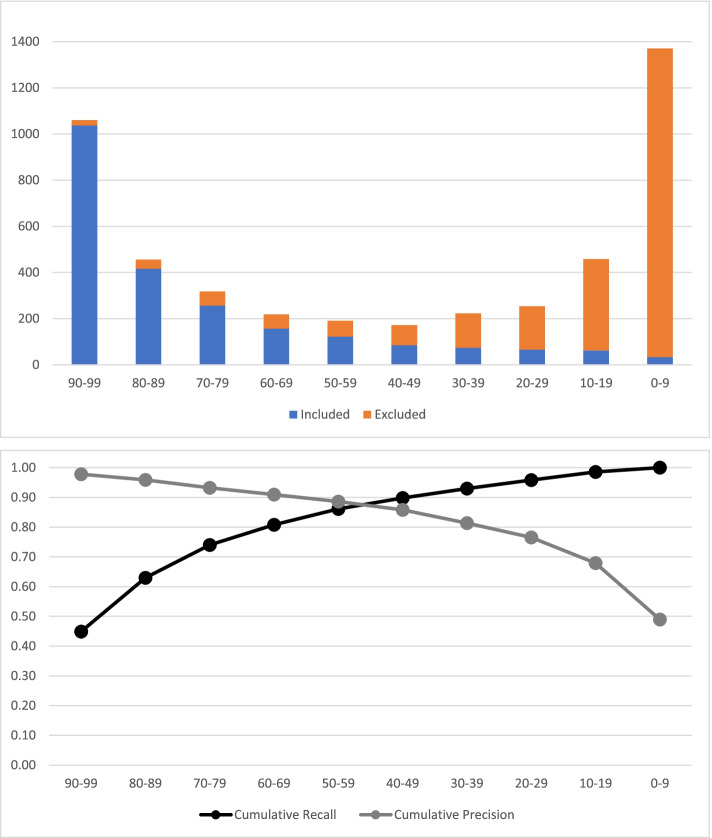
Distribution of classifier scores among ‘included’ and ‘excluded’ evaluation records (*N*=4722) and related performance metrics

Evaluation results for the classifier are shown in Fig. 2 and Table 2. In the evaluation data set, retaining records scoring at or above the calibrated threshold score would have resulted in 0.99 recall among ‘included’ records, with an overall workflow precision of 0.64 and a corollary 24.1% reduction in manual screening workload.

**Table 2:** Distribution of classifier scores among ‘included’ and ‘excluded’ evaluation records and related performance metrics

Classifier Score	90–99	80–89	70–79	60–69	50–59	40–49	30–39	20–29	10–19	0–9	Totals
**Included** ***N***	1037	417	256	157	122	85	74	66	63	33	2310
**Excluded** ***N***	23	39	62	62	69	87	149	188	395	1338	2412
**Totals**	1060	456	318	219	191	172	223	254	458	1371	
**Precision**	0.98	0.91	0.81	0.72	0.64	0.49	0.33	0.26	0.14	0.02	
**Cumulative recall**	0.45	0.63	0.74	0.81	0.86	0.90	0.93	0.96	0.99	1.00	
**Cumulative precision**	0.98	0.96	0.93	0.91	0.89	0.86	0.81	0.77	0.68	0.49	0.98
**Threshold classifier score**	7										
**Screened included** ***N***^a^	2285										
**Screened excluded** ***N***^a^	1299										
**Precision**	0.64										
**Discarded (‘Lost’) included** ***N***^a^	25										
**Discarded excluded** ***N***^a^	1113										
**Recall**	0.99										
**Net workload reduction** ***N***^a^	1138										
**Net workload reduction %**^a^	24.1%										

^a^At threshold score = 7

In our analysis of the 25 (1%) ‘missed’(discarded) ‘included’ records, we found that 12 were title-only records. Of these, four were errata or replies to studies already included in the CCSR and were therefore not the primary reference to the study. All but one of the ‘missed includes’ had been sourced from PubMed. Only two were records of interventional studies, the rest were records of observational studies. One ‘missed’ interventional study was an RCT, but it was not reporting the results of the RCT. The other one was a single-arm study that was not about COVID-19 patients, but the broader impact of the pandemic on the mental health of students, and the effects of a mindfulness component of the intervention described. Of the remaining ‘missed’ observational studies, most were studies looking at the broader impact of the pandemic on health services or selected populations. Three were small case-control or cohort studies that were diagnostic or prognostic in their aims. The remining three ‘missed’ records were all studies concerned with virus mutations. It is likely that this kind of study was not part of our stage 1 (training) data set, which contains studies from the first few months of the pandemic.

### Post hoc analysis of data set key characteristics

Results from comparing key characteristics between data sets used in the training, calibration and evaluation of the COVID-19 Study Classifier are shown in Table 3. Stage 1 (training) and Stage 2 (calibration) data sets were very similar in terms of the proportion of ‘included’ records in each set (35% and 37%, respectively). The Stage 3 (evaluation) data set, compiled of records manually screened for the CCSR during January 2021, had a higher proportion of ‘included’ records, at almost 50%. Each data set included a substantial proportion of title-only records (i.e. records without abstracts). The Stage 1 data set had the largest proportion of such records: 18,669 records (31%), of which 4495 were includes. Datasets 2 and 3 and a lower, but similar, proportion of title-only records: 23% and 19%, respectively.

**Table 3:** Key characteristics of development, calibration and evaluation data sets

Data set (classifier development stage)	Size	Number of eligible records (%)	Number of title-only records (%)	Number of title-only records that were eligible (%)	Provenance of records
**Data set 1 (Training)**	59,513	20,878 (35.1%)	18,669 (31.4%)	4495 (21.5%)	3229 (5.4%)—Embase 2083 (3.5%)—preprint 54201 (91.1%)—PubMed
**Data set 2 (Calibration)**	16,123	6005 (37.2%)	3626 (22.5%)	821 (13.7%)	1994 (12.4%)—Embase 287 (1.8%)—pre-print 13842 (85.8%)—PubMed
**Data set 3 (Evaluation)**	4722	2310 (48.9%)	896 (19.0%)	285 (12.3%)	89 (1.9%)—Embase 202 (4.3%)—pre-print 4431 (93.8%)—PubMed

## Discussion

We developed a binary ML classifier with the aim of reducing screening workload for the CCSR. Calibrated to achieve 99% recall, the classifier reduced screening workload by 24.1% in the evaluation data set. This finding was especially encouraging given the proportion of eligible records in this data set was close to 50%; and almost one in five of the records were ‘title-only’, with relatively few text features for classification, compared to records with accompanying abstracts. Title-only records in the context of the COVID pandemic can be resource- and time-intensive to manually assess. For many, more information will need to be found before a judgement on whether the record is eligible can be made. Having a classifier able to reliably reject ineligible title-only records is valuable and will free up human resource to assess the more unclear title-only records.

One of the main strengths of this study is the quality of the three data sets. We were able to use highly representative records for each stage, with a high level of confidence in the quality of each, derived as they were from the Cochrane Centralised Search Service team and Cochrane Crowd [7]. In addition, the training data set was fairly large (*n*=59,513), made up of both the class of interest (‘included’) and non-eligible records (‘excluded’). Records within the class of interest set encompassed all eligible study types (observational, interventional, qualitative and modelling studies) and designs and had good coverage across the range of possible study aims.

A potential limitation is that most records comprising each of the three study data sets were sourced from PubMed (of which a large proportion are also likely to have been indexed in Embase). This is unlikely to be an issue when applying the classifier to bibliographic records of journal articles identified from other database sources; but caution would be needed when applying the classifier to records with a different structure, for example, trial registry records. While many trial registry records contain similar information to a standard bibliographic record that could, in principle, be parsed and added to the title-abstract records prior to their classification, it is important to be aware of which fields map well to each other across the different record types, and in some cases to exclude certain fields of information that might confuse the classifier—such as trial exclusion criteria. As such, further work would be needed to evaluate the performance of this classifier when applied to records incorporating selected text from trial registry records. We could also investigate the potential to incorporate such records into sets used to retrain and recalibrate periodically updated versions of this classifier.

In this paper, we have focused on reporting the deployment of a machine learning classifier in a real-world scenario over a short period of time. The method employed, using train, test and calibration data sets and easily interpretable probabilities from a logistic regression classifier, provides a robust basis for future work and has proved acceptable to Cochrane. A workload reduction of ~25% is substantial given the high recall that must be achieved. However, we do not rule out that deployment of more sophisticated machine learning classification algorithms may be able to push the reported savings in workload marginally higher.

Evolution in the scope, aims and topics and text features of COVID-19 research over time suggest that ML classifiers which, like this one, that have been prospectively developed, are likely to need to be periodically retrained, recalibrated and re-evaluated, in order to minimise the risk of ‘losing’ (or ‘missing’) new bodies (or ‘strands’) of relevant research, with new ‘previously unseen’ text features, that are likely to emerge as the pandemic continues to unfold. Periodically updated training, calibration and evaluation data sets should be prospectively assembled to comprise records from three consecutive time periods, as we have done in the current study. This approach is robust in terms of its external validity, as it is consistent with the real-world use scenario in which such classifiers are deployed, where we do not know in advance how the research literature will evolve following their (re-) deployment. (Re-)calibrating and (re-)evaluating the classifier using records from consecutive time periods immediately succeeding the one covered by records in the

(re-)training dataset therefore confers further confidence (alongside the size and breadth of our study datasets) that any subtle evolution or ‘shifts’ in the scope and text features of bibliographic records of published COVID-19 research over time are unlikely to adversely impact on the performance of the deployed classifier in the short-term.

In late January 2021, the classifier developed in this study was deployed in the Cochrane COVID-19 register workflow, with records retrieved from PubMed and Embase.com being run through it. Workload reduction in terms of screening effort has been reduced in practice by approximately 20–25%, which is in line with the expected reduction based on this study. The classifier is also being used to help prioritise screening by ordering the records that score above the cut-point from highest to lowest score. Feedback from the screening team has indicated that records that receives high scores are almost always eligible studies, but they are often not the higher priority interventional studies. This is very likely due to the high prevalence of observational studies in the data sets used.

### Next steps

The Cochrane COVID-19 Study Classifier reduces screening burden by cutting the number of excludes to assess by approximately half. This is a helpful start but with the proportion of records eligible being around 50% (as it has been for the last 6 months for the CCSR), an ‘exclusion’ classifier can only do so much. In addition, the rate of publication on COVID-19 shows no sign of slowing with the number of new studies identified for the CCSR averaging 4600 per month over the last 6 months. Therefore, we are now developing additional automated approaches to maintain the CCSR. With over 60,000 COVID-19-related studies identified and tagged in the register, we are developing additional ML classifiers that will assign or suggest both study design characteristics and study aims to potentially eligible studies. We are also developing automated approaches to assigning PICO characteristics to interventional studies. Here, we will use crowd and ML capabilities in a hybrid approach to keeping up with the deluge of publications on COVID-19.

## Conclusions

The Cochrane COVID-19 Study Classifier can reduce manual screening workload for identifying COVID-19 research studies, with a very low and acceptable risk of missing eligible studies. This classifier is now deployed in the study identification workflow for the Cochrane COVID-19 Study Register.

## Data Availability

All data used in this analysis are available here: https://github.com/EPPI-Centre/CochraneCOVID19Classifier

## Abbreviations

CCSR: Cochrane COVID-19 Study Register
ML: Machine learning
RCT: Randomised controlled trial
